# Effects of sevoflurane and propofol on the optic nerve sheath diameter in patients undergoing laparoscopic gynecological surgery: a randomized controlled clinical studies

**DOI:** 10.1186/s12871-021-01243-7

**Published:** 2021-01-27

**Authors:** Weilian Geng, Changxing Chen, Xingfeng Sun, Shaoqiang Huang

**Affiliations:** 1 Department of Anesthesia, Obstetrics and Gynecology Hospital of Fudan University, No.128, Shenyang RD, Yangpu district, Shanghai, 200090 China; 2 Department of Emergency and Critical Care Medicine, Shanghai General Hospital, Shanghai Jiao Tong University School of Medicine, Shanghai, China

**Keywords:** Optic nerve sheath diameter, ONSD, sevoflurane, Propofol, CO_2_ pneumoperitoneum, Trendelenburg position

## Abstract

**Background:**

The results of studies on changes in intracranial pressure in patients undergoing laparoscopic surgery are inconsistent. Meanwhile, previous neurosurgery studies have suggested that propofol and sevoflurane have inconsistent effects on cerebral blood flow and cerebrovascular self-regulation. The purpose of this study is to compare changes in the optic nerve sheath diameter in patients undergoing laparoscopic gynecological surgery under anesthetic maintenance with propofol versus sevoflurane.

**Methods:**

This study included 110 patients undergoing laparoscopic gynecological surgery with an estimated operative time of more than 2 h under general anesthesia. The study was a randomized controlled study. The optic nerve sheath diameter (ONSD) at various time points was measured by ultrasound, including when the patients entered the operating room (Tawake), after successful anesthesia induction and endotracheal intubation (Tinduction), when the body position was adjusted to the Trendelenburg position and the CO_2_ pneumoperitoneum pressure reached 14 mmHg, which was recorded as T_0_. Then, measurements were conducted every 15 min for the first 1 h and then once every hour until the end of the surgery (T_15_, T_30_, T_45_, T_1h_, T_2h_ …), after the end of surgery and the tracheal tube was removed (T_end_), and before the patients were transferred to the ward (T_pacu_).

**Results:**

A significant difference in optic nerve sheath diameter was found between two groups at T_15_, T_30_, T_45_ (4.64 ± 0.48 mm and 4.50 ± 0.29 mm, respectively, *p* = 0.031;4.77 ± 0.45 mm and 4.62 ± 0.28 mm, respectively, *p* = 0.036;4.84 ± 0.46 mm and 4.65 ± 0.30 mm, respectively, *p* = 0.012), while there was no significant difference at T_awake_ and other time points.

**Conclusion:**

During laparoscopic gynecological surgery lasting more than 2 h, the optic nerve sheath diameter was slightly larger in the propofol group than that in the sevoflurane group in the first 45 min. No significant difference was observed between the two groups 1 h after surgery.

**Trial registration:**

clinicaltrials.gov, NCT03498235. Retrospectively registered 1 March 2018.

The manuscript adheres to CONSORT guidelines.

## Background

During laparoscopic gynecological surgery, due to the CO_2_ pneumoperitoneum and the steep Trendelenburg position (with the head lowered 45 degrees and the feet raised), cerebral venous recirculation becomes obstructed, and cerebral venous pressure increases. Meanwhile, intra-abdominal pressure increases, cerebrospinal fluid (CSF) absorption decreases, and intracranial pressure increases [1, 2]. The CO_2_ pneumoperitoneum causes hypercapnia, cerebrovascular dilation, increased intracranial cerebral blood flow, and increased intracranial pressure [3]. Although these effects rarely result in serious neurological complications such as cerebral haemorrhage and cerebral oedema [4], mild neurological complications, such as nausea, vomiting, and headaches, occur sometimes [5].

The results of different studies on changes in intracranial pressure in patients undergoing laparoscopic surgery are not consistent. Kim et al. compared patients undergoing laparoscopic gynecological surgery and laparoscopic gallbladder surgery under desflurane anesthesia and found that the pneumoperitoneum can cause a slight increase in intracranial pressure, but body position did not affect intracranial pressure, and intracranial pressure quickly returned to normal [6]. In a study of patients undergoing robot-assisted laparoscopic prostate surgery under sevoflurane anesthesia, Verdonck et al. found that optic nerve sheath diameter (ONSD) remained unchanged throughout the perioperative period [7].

Propofol and sevoflurane are commonly used anesthetic drugs. Previous neurosurgery studies have suggested that the two drugs have inconsistent effects on cerebral blood flow and cerebrovascular self-regulation [8]. Propofol dose-dependently contracts cerebral blood vessels, inhibits the cerebral oxygen metabolic rate, and reduces intracranial pressure [9, 10] but does not affect self-regulation of cerebral blood flow or the responsiveness of cerebral blood vessels to CO_2_ [11, 12]. Unlike propofol, the effect of sevoflurane on cerebral blood vessels depends on the balance between the direct vasodilating effect and the vasoconstricting effect caused by the reduction in cerebral metabolism [13]. Meanwhile, sevoflurane at a minimum alveolar concentration (MAC) of 0.5–1.5 does not affect self-regulation of cerebral blood flow or the reactivity of cerebral blood vessels to CO_2_ [14, 15]. It is unclear whether different anesthetic drugs have different effects on intracranial pressure because of the postural position and CO_2_ pneumoperitoneum in laparoscopic gynecological surgery.

The optic nerve sheath is a continuation of the cerebral dura mater with a transverse subarachnoid space, and its cerebrospinal fluid is also connected to the intracranial subarachnoid space. Therefore, when intracranial pressure increases, ONSD increases [16]. By artificially changing intracranial pressure, Hansen et al. [17] found that there is positive correlation between intracranial pressure and ONSD. Maissan et al. [18] believed that the ONSD could reflect changes in intracranial pressure in real time.

Compared with invasive intracranial pressure measurement, the ONSD measured by ultrasound is simpler, non-invasive, and convenient for bedside examination, and changes in intracranial pressure can be observed at any time [18, 19]. The purpose of this study is to compare the effects of propofol and sevoflurane on ONSD in patients undergoing laparoscopic gynecological surgery.

## Methods

This is a randomized controlled clinical trial. The ethics committee of Obstetrics and Gynaecology Hospital of Fudan University approved this study. The study was registered with clinicaltrials.gov (NCT03498235). A total of 110 patients who were classified as class I-II according to the standards and guidelines of the American Society of Anaesthesiologists (ASA) and underwent elective laparoscopic gynecological surgery under general anesthesia for an estimated operative time > 2 h from February 2018 to June 2020 were included in the study. The patients were randomly divided into the propofol group (Group P) or the sevoflurane group (Group S). Patients were randomized in a 1:1 ratio occurred by computerized sequence generation. An anesthesiologist, who was not involved in the study, created sealed opaque envelopes in which groupings were written randomized. Envelopes were opened in sequential order only after a patient had signed the consent form. The exclusion criteria were as follow: operative time < 2 h; body mass index (BMI) < 18.5 kg/m^2^ or ≥ 24 kg/m^2^; liver or kidney disease or abnormal results for related laboratory tests (C-reactive protein, hemoglobin, electrolytes, liver and kidney function, international normalized ratio, etc.); neuromuscular disease; allergies to anesthetics; pregnancy; and ophthalmological diseases.

The patients did not receive any preoperative drugs and were routinely monitored for non-invasive blood pressure, electrocardiography, and oxygen saturation. All patients received propofol, sufentanil 0.5 μg/kg and cisatracurium 0.1 mg/kg by intravenous injection for anesthesia induction and endotracheal intubation. TCI system was used for propofol, the target concentrations of propofol during anesthesia induction and maintenance were 4 μg/ml and 3.2 μg/ml, respectively. After successful intubation, mechanical ventilation was initiated in volumetric control mode, with a tidal volume of 6–8 ml/kg and a respiratory rate of 10–12 breaths/minute while no PEEP in all patients. The tidal volume and respiratory rate were adjusted to maintain an end-tidal CO_2_ of 35–40 mmHg. In the sevoflurane group, sevoflurane was maintained at 1–1.5 minimal alveolar concentration (MAC) in 50% oxygen/ air. Remifentanil at 0.25 μg·kg^-1^·min^-1^ and intermittent cisatracurium injections were used for anesthetic maintenance. The infusion rate of propofol or the concentration of sevoflurane was adjusted according to a Bispectral index (BIS) of 40–60. Thirty minutes before the end of the surgery, ondansetron was administered to prevent postoperative nausea and vomiting, and 4 mg of oxycodone was administered intravenously to relieve postoperative pain. The medications were discontinued immediately upon completion of the surgery. When the patient was awake, the tidal volume was greater than 6 L/min, and the respiratory rate was 14 ~ 20 breaths/min with no PEEP; the endotracheal tube had been removed. Afterwards, the endotracheal tube was removed, the patient was routinely monitored in the post-anesthetic recovery room (PACU) for 1 h.

If the intraoperative mean arterial pressure (MAP) was lower than 90 mmHg or decreased by > 30% from the baseline value, then a bolus of 100 μg of phenylephrine was administered. If the heart rate was less than 50 beats/minute, then 0.5 mg of atropine was administered. The angle of the Trendelenburg position adopted in the operation was 30°, and the CO_2_ pneumoperitoneum pressure was maintained at 14 mmHg. Patients were excluded from analysis due to intraoperative changes in surgical methods, such as conversion to vaginal surgery or transabdominal surgery, subcutaneous carbon dioxide emphysema development intraoperatively, and intraoperative changes in the anesthetic maintenance drugs.

Ultrasound (SonoSite M-Turbo, USA) was used for ONSD measurement. The patient assumed the supine position with the head in the middle position and the eyes gently closed. A disposable transparent patch was used to protect patient’s eyes. An ultrasound-coupling agent was evenly applied to both eyes and the ultrasonic probe. The 6-15 Hz high-frequency ultrasonic probe was gently placed above the upper eyelids without applying pressure to the globe. On the ultrasound screen, we can see a “long strip” hypoechoic area, which is perpendicular to the eyeball. The sheath structure with high echo can be seen at the edge of hypoechoic area. ONSD refers to the distance between the high echo sheath structures. The ONSD was measured at 3 mm behind the lateral edge of eyeball.

The images of left and right eyes were obtained three times separately at one time point, and all images were stored in DICOM and jpeg formats. A trained anaesthesiologist who was blinded for group allocation took the images of optic nerve sheath in all patients in this study, and ONSD was measured based on stored images by an experienced ultrasound doctor, and the average value was taken, with an accuracy of 0.1 mm. Previous studies have suggested that no significant difference in ONSD exists between different surveyors [20], and trained doctors can also accurately measure the ONSD by ultrasound at the bedside [21].

The primary outcome is to compare the effects of propofol and sevoflurane on ONSD at different time points. The time points at which the ONSD was ultrasonically measured were when the patients entered the operating room (T_awake_), after anesthesia induction and endotracheal intubation (T_induction_), and when the body position was adjusted to the Trendelenburg position and the CO_2_ pneumoperitoneum pressure reached 14 mmHg, which was recorded as T_0_. Then, the ONSD was measured every 15 min for the first hour followed by every hour until the end of the surgery (T_15_, T_30_, T_45_, T_1h_, T_2h_…), after anesthesia and drug discontinuation and extubation (T_end_), and immediately before transfer from the anesthesia recovery room to the ward (T_pacu_). Each time that the ONSD was measured by ultrasound, MAP and BIS was recorded.

### Statistical analysis

The quantitative data with a normal distribution were expressed as the mean ± standard deviation, while the quantitative data with a non-normal distribution were expressed as the median (interquartile range, IQR). Analysis of variance was conducted on repeated measurement data within the groups, and the Student-Newman-Keuls (SNK) q test was used for comparisons between the two groups. *P* values were adjusted by Bonferroni correction. *P* < 0.05 was considered statistically significant.

### Calculation of sample size

The early stage of sample size calculation included 15 female patients in the preliminary experiment. The standard deviation of ONSD preoperatively when the patients were conscious was 0.42 mm. According to the research results of Hansen et al. [17], every 1-mmHg increase in intracranial pressure corresponds to a 0.025-mm increase in the ONSD. Consistent with the study of Robba et al. [22], we believe that variation in the ONSD greater than 0.25 mm is clinically significant. At the levels of α = 0.05 and β = 0.1, the sample size of each group was calculated to be at least 48 cases. Considering the likelihood that approximately 25% of the patients would withdraw from the study, 60 cases were needed for each group.

## Results

**Fig. 1:**
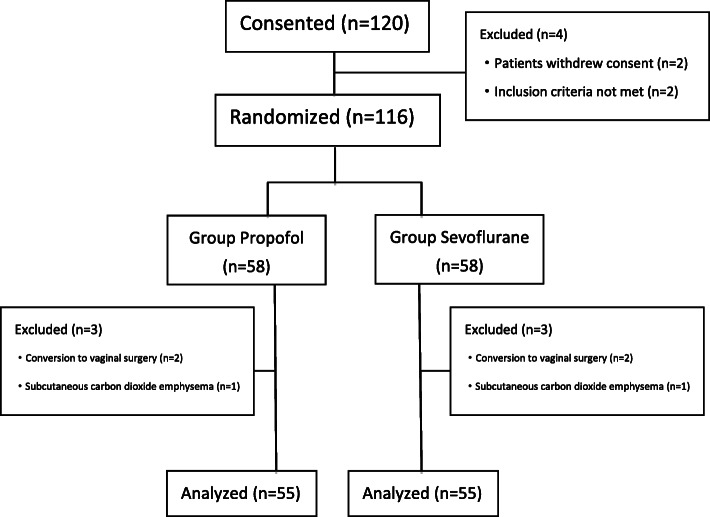
Flow diagram

Among the 120 female patients who underwent elective laparoscopic gynecological surgery, due to not meeting inclusion criteria or refusing to participate, 116 patients were included in this study, with 58 patients in each group. Due to CO_2_ pneumoderma or changes in surgical methods, 55 cases in each group were finally analysed (Fig. 1). The general conditions of the patients are shown in Table 1.

**Table 1:** Baseline Characteristics

	Group P (*n* = 55)	Group S (*n* = 55)	*P*
Age (y)	40.53 ± 11.08	41.15 ± 10.26	0.762
Height (cm)	161.18 ± 4.20	159.79 ± 4.50	0.097
Weight (kg)	59 (54.5, 63)	56 (51.9, 60)	0.057
BMI (kg/m2)	22.74 ± 2.28	22.31 ± 2.15	0.262
Surgery duration (h)	2.33 (2.12, 2.75)	2.5 (2.22, 2.75)	0.165
Total blood loss (ml)	100 (50, 120)	80 (50, 100)	0.424
Urine volume (ml)	400 (300, 500)	400 (400, 500)	0.088
Fluid volume (ml)	1200 (1100, 1500)	1200 (1100, 1500)	0.645
Airway pressure (mmHg)	15 (13, 16)	15 (13, 16)	0.754

The quantitative data with a normal distribution were expressed as mean ± standard deviation.

The quantitative data with a non-normal distribution were expressed as median (interquartile range, IQR)

*BMI* Body mass index

**Fig. 2:**
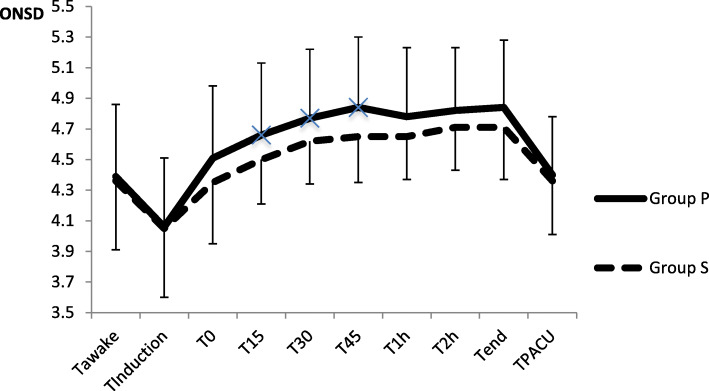
Comparison of ONSD at each time points between two groups. × meant there were statistical differences between two groups at time points T_15_, T_30_ and T_45_ (Values of p were 0.031, 0.035 and 0.028). Comparison of ONSD at other time points, values of p were 0.984, 0.666, 0.646, 0.065, 0.211, 0.368 and 0.646 at T_awake_, T_induction_, T_0_, T_1h_, T_2h_, T_end_ and T_pacu_

The comparison of the ONSD at each time point between the two groups is shown in Table 2 and Fig. 2. No significant difference in the baseline preoperative ONSD was found between the two groups. After anesthesia induction, the ONSD values all decreased compared to the baseline value in both two groups, but no significant differences between the two groups. At three time points (T_awake_, T_induction_, and T_0_), the ONSD was not significantly different between the two groups (*p* = 0.984, 0.666, and 0.646, respectively). Over time, at three time points (T_15_, T_30_, and T_45_), significant differences in ONSD were identified between the two groups (*p* = 0.031, 0.035, and 0.028, respectively). At T_1 h_, T_2 h_, T_end_, and T_pacu_, no significant differences in ONSD were found between the two groups after statistical correction (*p* = 0.065, 0.211, 0.368, and 0.646 respectively).

**Table 2:** Comparison of ONSD at different time points between two groups

	Group P (*n* = 55)	Group S (*n* = 55)	*P*
ONSD T_awake_ (mm)	4.39 ± 0.37	4.36 ± 0.45	0.694
ONSD T_Induction_ (mm)	4.06 ± 0.45^*^	4.05 ± 0.45^*^	0.882
ONSD T_0_ (mm)	4.53 ± 0.47^*#^	4.35 ± 0.40^*#^	0.058
ONSD T_15_ (mm)	4.64 ± 0.48^*#^	4.50 ± 0.29^*#^	0.031
ONSD T_30_ (mm)	4.77 ± 0.45^*#^	4.62 ± 0.28^*#^	0.036
ONSD T45 (mm)	4.84 ± 0.46^*#^	4.65 ± 0.30^*#^	0.012
ONSD T_1h_ (mm)	4.83 ± 0.43^*#^	4.66 ± 0.28^*#^	0.066
ONSD T_2h_ (mm)	4.82 ± 0.41^*#^	4.71 ± 0.28^*#^	0.089
ONSD T_end_ (mm)	4.84 ± 0.44^*#^	4.71 ± 0.34^*#^	0.082
ONSD T_PACU_ (mm)	4.40 ± 0.38^*#^	4.36 ± 0.35^*#^	0.641

Values were expressed as the mean ± standard variation. * meant comparison of ONSD intra group at each time points to T_awake._ In Group *P*, values of p were 0.000 except comparison of T_pacu_ to T_awake_ which p value was 0.945; in Group S, values of p were 0.939、0.035 and 0.884 when compared T_0_, T_15_ and T_pacu_ to T_awake_, while *p* values were 0.000 at any other time points.# meant comparison of ONSD intra group at each time points to T_induction_, and all *p* values were 0.000 in both two groups

**Fig. 3:**
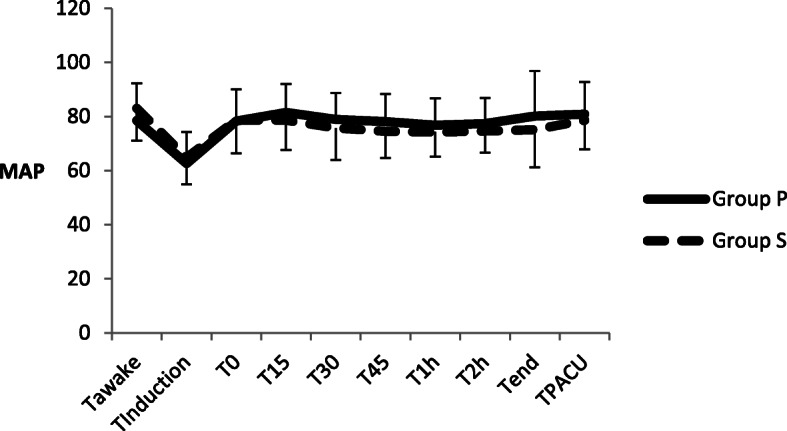
Comparison of MAP at each time points between two groups. There are no significant differences between two groups at each time points (Values of p were 0.066, 0.312, 0.912, 0.156, 0.125, 0.064, 0.166, 0.095, 0.092 and 0.290). MAP Mean arterial pressure

**Fig. 4:**
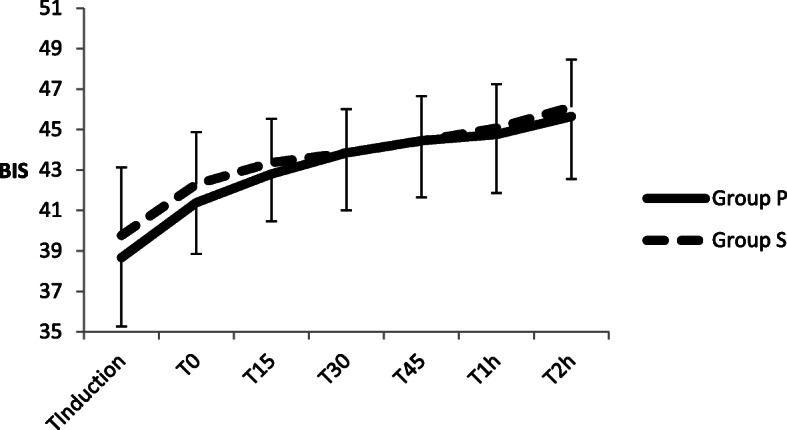
Comparison of BIS at each time points between two groups. There are no significant differences between two groups at each time points (Values of p were 0.093、0.065, 0.191, 1.000, 0.970, 0.503 and 0.368). BIS Bispectral index

The comparison of the MAP and BIS at each time point between the two groups is shown in Fig. 3 and Fig. 4.There are no significant differences in MAP and BIS at each time point between Group P and Group S. No hypotension or serious neurological complications such as cerebral haemorrhage or cerebral oedema occurred in either group.

## Discussion

This study compared the effects of two general anesthesia drugs (propofol and sevoflurane) on the ONSD in patients undergoing laparoscopic gynecological surgery. The results showed that the ONSD was significantly reduced compared to the baseline value in the patients in the two groups after anesthesia induction and endotracheal intubation. With establishment of the CO_2_ pneumoperitoneum and the Trendelenburg position, the ONSD in both groups increased and exceeded the baseline value. However, at first 45 min, the amplitude of the increase in the propofol group was greater than that in the sevoflurane group. Over time, considering the pneumoperitoneum and the Trendelenburg position, no significant difference in the ONSD was found between the two groups from 1 h after starting the surgery to extubation at the end of the surgery. When leaving the recovery room, the ONSD returned to baseline in the patients in both groups.

In this study, both groups received propofol and sufentanil for anesthesia induction, and cisatracurium was used for endotracheal intubation. Both sufentanil and propofol contract cerebral vessels and reduce the cerebral metabolic rate [9, 10, 23]; therefore, the ONSD after anesthesia induction was reduced compared to the baseline value.

With establishment of the CO_2_ pneumoperitoneum and the Trendelenburg position, the ONSD increased gradually but recovered to baseline by 1 h after surgery. The reason may be that the effect of the CO_2_ pneumoperitoneum and body position on intracranial pressure exceeds the effect of drugs on intracranial blood flow. Bilateral internal cervical vessels and vertebral vessels play an important role in cerebral circulation [24]. After establishment of the CO_2_ pneumoperitoneum and the Trendelenburg position, recirculation in the internal jugular vein and vertebral vein was obstructed. At the same time, the mean arterial pressure during establishment of the pneumoperitoneum and positioning was elevated compared to that after anesthesia induction. Whiteley et al. found that the ONSD was positively correlated with the mean arterial pressure [25].

For the propofol group, our results are basically consistent with those of Blecha et al. [26]; however, the amplitude of changes in the optic nerve sheath was higher than that in the study of Blecha et al., and maybe the reason for the difference is that the patients in the study of Blecha et al. received midazolam before surgery, which can reduce intracranial pressure. In addition, the patients in that study were from western countries, and the ONSD varied among different races. Wang et al. found that among the Chinese population, the predicted cut-off value of ONSD that means intracranial pressure higher than 20 cmH_2_O was lower than that in Caucasians [27].

The effects of CO_2_ pneumoperitoneum and Trendelenburg position establishment on the ONSD in sevoflurane versus propofol anesthesia are different in various studies. We found that although the ONSD increased in the sevoflurane group, the amplitude of the increase was smaller than that in the propofol group at the early stage of surgery. In the study of Robba et al. [22], the amplitude of the increase in the ONSD after sevoflurane anesthesia was consistent with that in our study. However, the studies of Kim et al. [28] and Chin et al. [29] showed that the amplitude of the increase in the ONSD after sevoflurane anesthesia was higher than that in our study. Verdonck et al. [7] believed that the ONSD remains unchanged in patients undergoing sevoflurane anesthesia.

The results of this study are generally consistent with those of Lee et al. [30], while in the first 30 min of operation, the results were inconsistent. The main reason may be that Lee et al. used midazolam and glinbromide before operation; on the other hand, study of Lee et al. only maintained BIS at 40–60 during the whole operation, but did not compare the BIS values between the two groups. The depth of anesthesia may affect the changes of cerebral blood flow, thus further affecting the changes of intracranial pressure and ONSD value.

The inconsistencies across different studies may be due to the lack of consistency in patients’ anesthetic depth and sevoflurane blood concentration. Sevoflurane has a dominant effect on cerebral oxygen metabolism at a low concentration; while at medium and high concentrations, it has a direct vasodilatory effect [31]. Propofol reduces cerebral blood flow more because of its effect on reducing cerebral oxygen metabolism rather than direct vasoconstriction [32, 33]. Meanwhile, the ONSD can reflect intracranial pressure in real time; however, the correlation coefficient between ONSD and intracranial pressure in previous studies was 0.660–0.820 [19, 34, 35]. Hansen et al. believed that the ONSD and intracranial pressure have an elastic nonlinear relationship [17]. In other words, the ONSD may better reflect changing trends in intracranial pressure than specific values.

We found that although the ONSD increased significantly in both groups, it returned to baseline 1 h after surgery. Animal studies suggest that with establishment of the CO_2_ pneumoperitoneum and the Trendelenburg position, intracranial pressure increased by 10 mmHg compared to the baseline value [36]. However, Kalmar et al. [37] believe that intracranial pressure fluctuations within the physiological range are regulated by multiple mechanisms, and that the intracranial pressure increases exponentially only when these regulatory mechanisms are exhausted. Notably, the brain has a strong ability to transfer CSF to the vascular system, and when intracranial pressure increased, CSF moved intrathecally at a rate of 2 ml/min [38, 39], which is also why the ONSD returned to baseline 1 h after surgery. On the other way, a reduction of the intracranial blood volume after termination of the steep Trendelenburg position could be the reason.

Four limitations exist in this study. First, this study did not analyse changes in the ONSD in patients with longer operative times (> 3 h), mainly because few cases required long operative times (> 3 h) in this study. Hansen et al. suggested that prolonged intracranial hypertension affected the reversibility of optic nerve sheath changes [17]. Therefore, we hypothesized that the duration of the increase in ONSD in patients undergoing prolonged laparoscopic gynecological surgery would be prolonged, but further studies are needed for confirmation. Second, all the patients included in this study were female patients younger than 65 years old, and further studies are needed to determine whether similar conclusions can be established for male or elderly patients. Third, in our study, sevoflurane was maintained at 1–1.5 minimal alveolar concentration (MAC) while we do not record values of end-tidal concentrations of sevoflurane (Etsevo). Animal studies suggest that cerebral blood flow may not be changed when Etsevo is 0.3–1.5MAC [40]. Artru et al. find ICP is not changed when sevoflurane is 0.5MAC, 1.0MAC or 1.5MAC in neurosurgery patients [41]. We guess that small changes in Etsevo during 0.5–1.5MAC may not have significant effects on cerebral blood flow. Fourth, Whiteley et al. suggest that ONSD is positively correlated with MAP [25]. In our study, there are no significant differences between Group S and Group P at different time points and that is why we ignore the effect of blood pressure on ONSD.

In conclusion, we found that when comparing the two drugs, at the early stage, the ONSD post-pneumoperitoneum in the propofol group was slightly larger than that in the sevoflurane group, and the difference was statistically significant. No significant difference was observed between the two groups 1 h after surgery.

## Data Availability

The trial protocol, datasets used and/or analysed during the current study are available from the corresponding author on reasonable request.

## Abbreviations

ONSD: Optic nerve sheath diameter
ASA: American Society of Anaesthesiologists
MAP: Mean arterial pressure
BIS: Bispectral index
MAC: Minimal alveolar concentration
PACU: Post-anesthetic recovery room
